# The Influence of Promoter Architectures and Regulatory Motifs on Gene Expression in *Escherichia coli*

**DOI:** 10.1371/journal.pone.0114347

**Published:** 2014-12-30

**Authors:** Mattias Rydenfelt, Hernan G. Garcia, Robert Sidney Cox, Rob Phillips

**Affiliations:** 1 Department of Physics, California Institute of Technology, Pasadena, CA, United States of America; 2 Integrative Research Institute for the Life Sciences and Institute for Theoretical Biology, Humboldt University, Berlin, Germany; 3 Joseph-Henry Laboratories of Physics, Princeton University, Princeton, NJ, United States of America; 4 Department of Chemical Science and Engineering, Kobe University, Kobe, Japan; 5 Department of Applied Physics, California Institute of Technology, Pasadena, CA, United States of America; 6 Division of Biology, California Institute of Technology, Pasadena, CA, United States of America; Universitat Pompeu Fabra, Spain

## Abstract

The ability to regulate gene expression is of central importance for the adaptability of living organisms to changes in their external and internal environment. At the transcriptional level, binding of transcription factors (TFs) in the promoter region can modulate the transcription rate, hence making TFs central players in gene regulation. For some model organisms, information about the locations and identities of discovered TF binding sites have been collected in continually updated databases, such as RegulonDB for the well-studied case of *E. coli*. In order to reveal the general principles behind the binding-site arrangement and function of these regulatory architectures we propose a random promoter architecture model that preserves the overall abundance of binding sites to identify overrepresented binding site configurations. This model is analogous to the random network model used in the study of genetic network motifs, where regulatory motifs are identified through their overrepresentation with respect to a “randomly connected” genetic network. Using our model we identify TF pairs which coregulate operons in an overrepresented fashion, or individual TFs which act at multiple binding sites per promoter by, for example, cooperative binding, DNA looping, or through multiple binding domains. We furthermore explore the relationship between promoter architecture and gene expression, using three different genome-wide protein copy number censuses. Perhaps surprisingly, we find no systematic correlation between the number of activator and repressor binding sites regulating a gene and the level of gene expression. A position-weight-matrix model used to estimate the binding affinity of RNA polymerase (RNAP) to the promoters of activated and repressed genes suggests that this lack of correlation might in part be due to differences in basal transcription levels, with repressed genes having a higher basal activity level. This quantitative catalogue relating promoter architecture and function provides a first step towards genome-wide predictive models of regulatory function.

## Introduction

One of the most impressive accomplishments in molecular biology over the past half-century has been the mapping of thousands of gene interactions to create genetic networks for a broad collection of organisms. Such maps have made it possible to qualitatively understand how groups of genes can together provide important functionality. Still, the genetic network descriptions leave us with a picture of the regulatory landscape that is not quantitatively predictive. Although impressive, genetic networks do not provide us with all the information necessary to make concrete predictions, such as the number of proteins produced of a given kind under particular environmental conditions, not the least because the notions of ‘activation’ and ‘repression’ are often inherently verbal rather than quantitative. The amount of activation or repression achieved by transcription factors (TFs) can vary by many orders of magnitude, depending on how tightly TFs bind to the promoter of interest [1] and many other factors. Moreover the resulting response curves depend on *promoter architecture*, i.e. the particular configuration of TF binding sites. For example, a repressor that blocks a promoter through DNA looping (e.g. LacI) has been shown to have a steeper response curve than its unlooped counterpart [2]. Furthermore, genetic networks do not tell if the TF that is supposed to regulate a gene is actually present in the cell at all, which might not be the case if it is inactivated through nucleosomal organization or by chromatin remodeling complexes [3], [4].

For genetic networks to be predictive tools in biology they need to be be augmented with quantitative descriptions of the census of regulatory players. Our goal with the present paper is to take a step in this direction by studying the role of promoter architectures in transcriptional regulation, from a genome-wide point of view. No organism offers a better opportunity to do so than *E. coli*, which after more than half a century of intense study demonstrates the most well understood regulatory network. Through ambitious efforts many cold and hard facts about transcriptional regulation in *E. coli* have been collected and made easily accessible in databases like RegulonDB [5] and EcoCyc [6]. These contain information including, but not limited to, which TFs regulate different operons, where they bind to promoters, and their regulatory effect (activation or repression). All of these features play an important role in transcriptional regulation. A TF which binds cooperatively to multiple binding sites, either through direct contact or DNA looping, provides a steeper regulatory response, typically reported by Hill coefficients, than TFs binding just a single site [7]. The position of binding sites play an equally important role. In experiments where a single repressor binding site has been systematically moved along the promoter region [8]–[11], the repression shows a clear dependence on position, interestingly featuring a 10–11 bp modulation following the periodicity of the DNA helix.

In this paper we study both the positions and multiplicities of TF binding sites in *E. coli*, for the 2500 or so known TF-DNA interactions in RegulonDB 8.5. A challenge inherent in using RegulonDB, EcoCyc, or any other biological database as primary information source is that the data is inevitably incomplete. More than half of the genes in *E. coli* still lack any regulatory annotation, including important genes such as those responsible for mechanosensation. We must therefore be cautious when interpreting our results. Whereas there is no obvious reason that, for example, binding site positions are biased, the absolute number of binding sites is almost certainly underestimated. This assertion is supported by the fact that the rate of newly discovered TF binding sites does not show any sign of slowing down, thanks to the advent of powerful techniques such as ChIP-seq [12] and Sort-Seq [13]. A healthy skepticism from the reader is thereby encouraged and the results should be viewed as provisional until more of the underlying regulatory facts are in hand.

We view the work presented here as a step towards using promoter architectures to give a more detailed understanding of transcriptional regulation than can be given by a genetic network map alone. Hopefully these findings can also provide valuable input for the theoretical dissection of transcription regulation, which has shown increasing capability to make distinct predictions for the response function of different promoter architectures [14]–[17]. Perhaps most importantly, the analysis presented here shows how far short the current factual understanding of regulatory architectures and measured expression levels falls from serving as a predictive framework, and thus should be seen as a call for higher predictive expectations and a more rigorous treatment of the relation between regulatory architecture and input-output functions.

## Models

### Random promoter architecture model

Following the classic method of random graphs [18], biological networks have been compared to randomly constructed networks to find *network motifs*, corresponding to recurring patterns in the connections between genes, which are overrepresented compared to a random graph [19]. One well-studied network motif is the feed-forward loop, where a single gene is regulated by two TFs, and in addition one of the TFs regulates the other. Network motifs are presumedly selected for in biological systems because of functionality they provide, for example, robustness against concentration fluctuations of regulatory molecules. We similarly use a random assignment of TFs to create a null model of promoter architecture, and identify overrepresented promoter architectures motifs deviating from this expectation. For this we need to introduce a *random promoter architecture model*, to be used as reference for identifying overrepresented reported promoter architectures.

TF binding sites can be both lost and gained due to the steady pace of mutations across the genome. If we assume that these mutations occur randomly and uniformly across the genome, then in the absence of selection any specific distribution of a given number of TF binding sites over a set of operons would be as probable as any other. If a certain class of promoter architecture occurs more frequently in real regulatory networks than in this null model, we expect them to encode biological functions which are advantageous. The simple approach we will adopt to implement a random promoter architecture is therefore to imagine all binding sites reported in RegulonDB as being “sprinkled” over all operons with *uniform* probability. The mathematical implications of this simple postulate will be developed here, saving for the Results section the task of identifying promoter architecture motifs and how they differ from this simple null model.

As a first application of the random promoter architecture model we will look at the distribution of number of binding sites per operon. Throughout this work we will consider binding sites for a given TF as indistinguishable even if they have a different DNA sequence. Additionally, we define an operon as a cluster of transcriptional units, containing one or more protein coding sequences and one or more promoters which initiate transcription in the same direction to create mRNAs which carry the protein coding sequences. While some transcriptional units express RNA with no protein coding sequences, we ignore these cases for the time being. For this model we also explicitly neglect cases where one binding site can regulate more than one operon. In fact, only a small number of binding sites are known to regulate multiple operons (9% in RegulonDB 8.5). With these assumptions we can describe the random model of promoter binding site architecture.

There are 2871 TF binding sites listed in RegulonDB 8.5, and 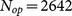 operons. We first consider the distribution of a single type of TF binding site with 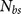 copies. The probability 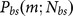 of a given operon to have exactly 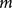 binding sites assigned is described by the binomial distribution
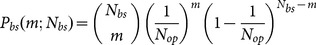
(1)

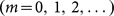



Here 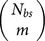 is the number of ways to choose a set of 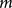 binding sites from the pool of 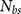 sites, 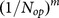 is the probability that they are all assigned to a given operon, and 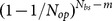 is the probability that the rest of the bindings sites are assigned to the other operons.

Since the probability is small for a binding site to be assigned a particular operon, namely 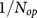, the binomial distribution can be approximated by a Poisson distribution with mean 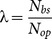. For the numbers of 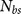 and 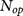 given by RegulonDB 8.5 the Poisson approximation is valid to within 1% for 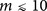, which covers 98% of the operons in the dataset. However, for very highly regulated operons the Poisson approximation should not be used.

We can generalize the distribution in to incorporate several types of binding sites [see Eq. (2)], say activators or repressors, or different particular TFs. Since all binding sites are assumed to be independently distributed, the probability distribution 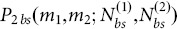 for an operon to end up with 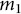 binding sites (from a total of 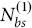) of one type and 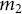 binding sites (from a total of 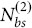) of a second type will be given by an independent product of two binomial distributions
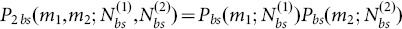
(2)

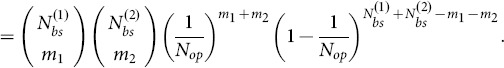
(3)


Several TFs in *E. coli* preferentially bind to multiple binding sites at a given promoter, for example NarP binds to two sites or more at 10 out of 11 regulated operons according to RegulonDB 8.5. Such examples of operator multiplicity can occur due to cooperative contacts between the protein copies, or due to other reasons such as multiple transcription start sites and other TF interactions. How can we rigorously define the level at which binding sites of a TF cooccur around a promoter? Simply looking at the absolute number of operons with multiple binding sites is not a good measure of clustering of a TF at a promoter, as it does not take into account the total number of binding sites available. A *global TF* [20], [21] with hundreds of binding sites will likely bind at multiple sites at several promoters simply by chance.

**Figure 1:**
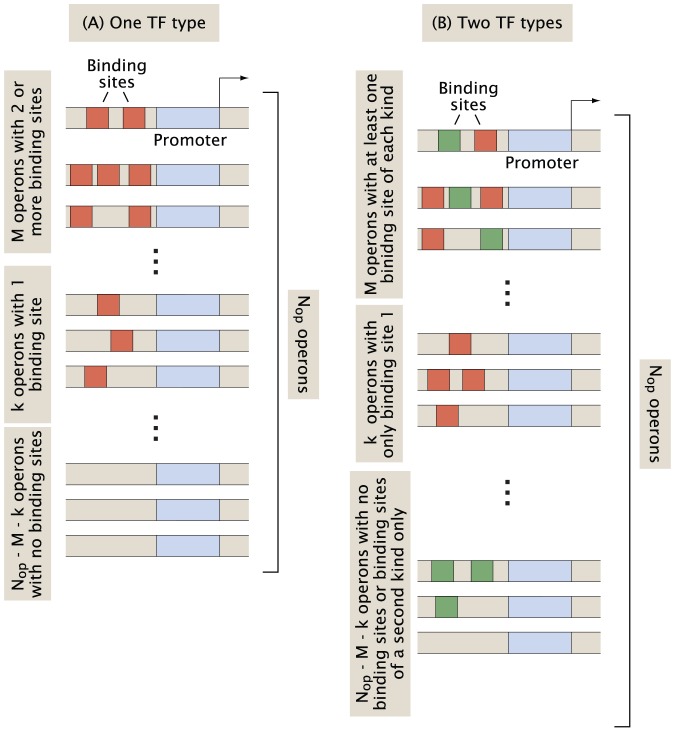
Combinatorics for distribution of binding sites across the genome. (A) *M* operons with at least two binding sites, and *k* operons with exactly one binding site. The remaining 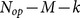 operons are empty. (B) *M* operons with at least one binding site of each kind, and *k* operons with at least one binding site of first kind but none of the second. The remaining 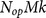 operons are either empty or have only binding sites of the second kind.



We use the random promoter architecture model to derive the probability 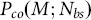 for 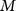 operons to be regulated by at least two binding sites for the same TF, as a function of the total number of binding sites 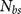 for that TF (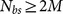). To find the number of ways 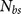 binding sites can be distributed over 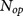 operons, with two or more sites at 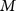 of these, we first choose 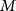 operons, in any of 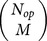 ways, and assign two binding sites to each of them. See illustration Fig. 1(A) for a schematic description of the model. Next we put 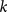 of the remaining 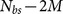 binding sites into 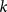 of the remaining 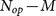 operons (i.e. one binding site per operon), which we can choose in 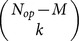 ways. Finally we put the remaining 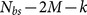 binding sites into the *M* operons, which already have two binding sites, however we want. The number of ways this can be done equals the number of nonnegative integer solutions to the equation 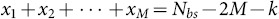, a famous problem from combinatorics which is equivalent to the number of ways of placing (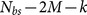) identical balls into 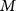 bins, with 
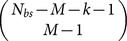
 solutions. To find the probability 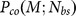 we sum over 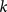 and divide by the total number of ways to distribute 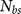 binding sites over 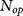 operons, which according to the same argument as above is given by 
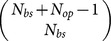
, resulting in (for 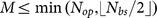)

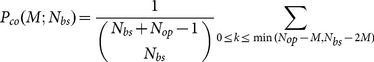
(4)

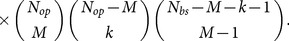
(5)


By using the continuous definition of binomial coefficient 
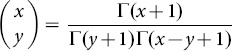
 where 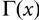 is the gamma function [22], Eq. (5) gives us the right probability also for 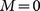, namely 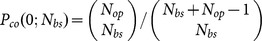 when 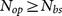, and 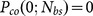 when 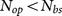.

We can generalize this problem and analyze whether binding sites of a pair of TFs tend to cluster together. TF pairs which coregulate operons more frequently than suggested by the random promoter architecture model are more likely to have related biological function. Let 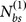 and 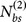 be the number of binding sites for two different types of TFs. As above we start by choosing 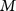 operons where we put one binding site of each kind. Next we put the remaining 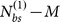 binding sites of first type into the 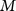 “shared” operons plus an additional of 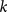 operons, which we can choose in 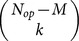 ways, with at least one binding site in each. Fig. 1(B) gives a schematic of this procedure. The number of ways this can be done equals the number of integer solutions to the equation below with the given constraints
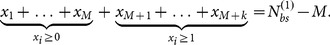
(6)


After subtracting 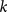 from both sides one realizes that the number of solutions to this equation equals the number of nonnegative integer solutions to the simpler Eq. (7), with 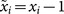,
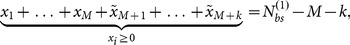
(7)


which is given by 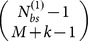. Next we distribute the remaining 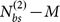 binding sites of the second kind onto any of the operons, except from the 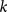 operons dedicated for binding sites of the first kind only. This can be done in 
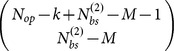
 ways. We find the probability 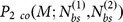 for 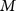 operons (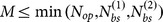) to be regulated by at least one binding site of each type by summing over 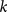 and dividing by the total number of binding site arrangements, which is given by 
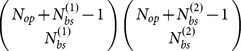
, resulting in
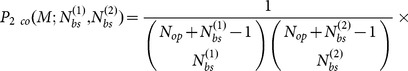


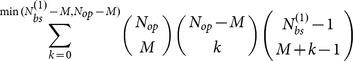


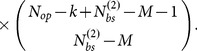
(8)


As a sanity check we use MATHEMATICA to see that the probabilities add up to one.

We can also compare with earlier work, which solved essentially the same problem but under the assumption that binding sites, even of the same kind, are distinguishable [23]. However, as long as the probability is small that two binding sites regulate the same operon the two methods will give similar results, just like Fermi-Dirac statistics approaches Boltzmann statistics in dilute systems [24].

A different method to identify TF cooperativity based on mutual information from ChIP data was used in [25]. The advantage of the random promoter architecture model is that it resolves biasing due to differences in number of TF binding sites, and allows us to determine both the expected number of coregulated operons and also the associated *p*-value of any given observation: i.e. the probability of an equal or more extreme outcome with respect to the random promoter architecture model. This will become useful in the Results section where we want to identify TF binding motifs in the reported distributions from RegulonDB.

### Linear energy model of RNAP-DNA binding

The binding affinity of RNAP to the promoter of a gene is determined by the nucleotide sequence of the promoter and has a strong influence on the transcription rate of the gene [17]. The more effectively a promoter can recruit RNAP and initiate open complex formation, the higher the transcription rate of the gene will be. Creating a predictive map between DNA sequence and RNAP binding affinity is a problem which has received much attention [13], [26]–[29]. One of the simplest but yet most successful approaches to the modeling of RNAP-DNA (or TF-DNA) interactions is to assume *independent* energy contributions from each individual nucleotide in the binding sequence. Under this linear assumption the total binding energy of a sequence 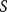 can be expressed as a simple matrix trace
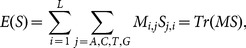
(9)


where 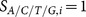 if the identity of the base at nucleotide position 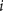 in the sequence is given by 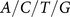 and otherwise 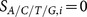, 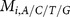 represents the energy contribution at position 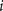 for base 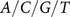 respectively, and 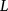 is the length (in base pairs) of the binding sequence.

Despite having considered all promoter types up until this point, we now focus our attention on promoters associated with the sigma factor 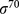, which is called the “housekeeping” sigma factor of *E. coli*. The RNAP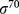 complex has two binding domains which may interact with the promoter at the −35 and −10 signals upstream of the transcription start site (+1) [30]. In this study we compute the binding energy from these two signals separately using the energy matrix 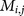 of Brewster et al. [26]. Starting with two position weight matrices for the two signals, we place the “−10 box” signal at the −9, −10, or −11 position and the “−35 box” signal 22–24 bp upstream from the −10 box. Thus we allow 9 possible configurations for each pair of −10 and −35 boxes, and choose the one with the lowest binding energy according to the reference energy matrix [26].

The commonly used *occupancy hypothesis* [9], [14], states a linear relationship between the transcription rate of a gene and the probability of its promoter being occupied by RNAP. This probability is, according to the Boltzmann distribution, proportional to 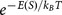 for systems in (quasi)equilibrium, an approximation which can be made if RNAP homogenizes throughout the cell at a much higher rate than that at which they are being produced. Despite its simplicity in ignoring details of open complex formation and promoter escape rate, the occupancy hypothesis has proved surprisingly successful in many different settings [1], [9], [26].

## Results

### How many genes do TFs regulate?

**Figure 2:**
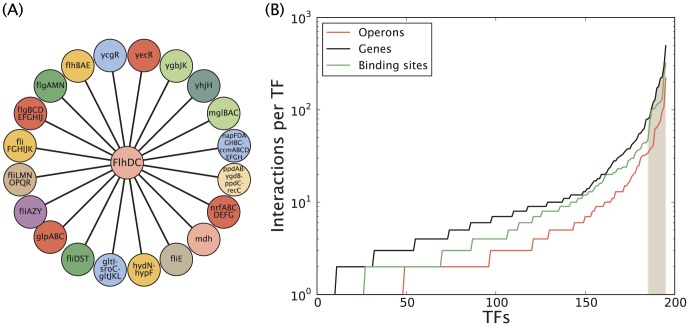
Number of operons, genes and binding sites regulated per TF (RegulonDB 8.5.) (A) Schematic of operons regulated by the FlhCD TFs according to RegulonDB 8.5. (B) The TFs have been sorted by increasing number of interactions, and the dark shaded area highlights the TFs responsible for 50% of all regulatory interactions in *E. coli*, which we denote as global TFs. The median number of operons, genes (coding sequences) and binding sites regulated per TF is 3, 4 and 6.5, respectively. The number of regulated genes is calculated by taking into account how many coding sequences are contained within each operon.

Genes of related biological function are often coregulated. For example a flagellum in *E. coli* consists of roughly forty different proteins [31] present at precise copy number ratios. Not all the flagellar genes are contained within the same operon, but instead these forty coding regions are transcribed from roughly ten different operons [6] (Fig. 2(A)). Coregulation of these operons allows the flagellar proteins to be expressed at precise ratios, a task which is handled by the TFs FlhC and FlhD in *E. coli*. However, other biological functions correlate with the production of flagella. For example production of sugar receptors in the cell membrane such as MglBAC, necessary for chemotaxis, are also regulated by FlhC and FlhD.

In general we expect “correlated genes” to be regulated by the same TFs, and the question we will address in this section is: How many correlated genes are typically regulated by the same TFs? In Fig. 2(B) we show the number of operons that are regulated by the same TF as reported by RegulonDB 8.5. By counting the number of coding sequences within each operon we also display the number of regulated genes per TF. Finally, in Fig. 2(B) we show the number of binding sites for each TF. The numbers provide a lower estimate of the actual *E. coli* regulatory network, acknowledging the fact that not all binding sites have yet been discovered. The figure reveals two almost separate groups of TFs: a large number of *specific TFs* which regulate only a few operons, and a mere handful of *global TFs* [20], [21] regulating up to a hundred operons [see Table 1] each. Half of all TFs regulate two operons or less, suggesting that, unlike the construction of flagella, many operons in *E. coli* are not strongly correlated and encode all of the proteins necessary for a particular phenotype. For example, in response to varying levels of copper in the cytoplasm ComR reportedly regulates only one single gene, *bhsA*, which alters the outer cell membrane permeability for copper [32]. Global TFs, which regulate core activities in the cell, for example metabolic pathways (e.g. CRP) or the rRNA of the translational machinery (e.g. Fis), are the exceptions to this rule. Despite the small number of global TFs, these are involved in roughly half of all reported regulatory interactions.

**Table 1:** Global TFs and their associated number of binding sites, the number of operons regulated, and the total number of genes (coding sequences) regulated by each TF (RegulonDB 8.5).

TF	Operons	Genes	Binding sites
CRP	221	495	320
FNR	108	296	131
Fis	96	225	237
IHF	76	219	114
H-NS	70	179	105
ArcA	64	172	118
Fur	63	129	122
Lrp	41	103	103

See S1 Table for a corresponding table that includes specific TFs. The notion of global TF is not unambiguously defined, and the list presented here might therefore differ slightly from that used in other works [90], [91].

**Figure 3:**
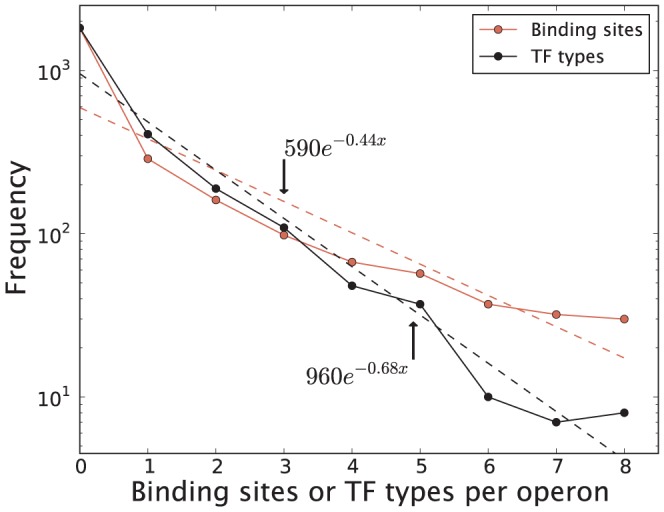
Number of binding sites and TF types regulating each operon (RegulonDB 8.5). The mean number of binding sites per operon is 1.1 (3.5 for operons with at least one known binding site). The best exponential fits (in log-space) are shown in the figure as dashed lines. These fits are expected to change as more binding sites are discovered.

To evaluate the regulatory complexity of a promoter, we can conversely consider the number of TFs regulating each operon. The more TFs regulating an operon, the more specific its response might be to various cellular conditions. Note that here we will consider operons regulated by any of the *E. coli* 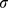 subunits. As a result, the numbers below are certain to change if restricting the analysis to 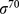. In Fig. 3 we show the number of TF interactions and number of different TF types regulating operons as reported by RegulonDB 8.5. The average number of TF binding sites per operon is only 1.1, but climbs to 3.5 when excluding operons without known regulatory interactions. This observation suggests that data in RegulonDB is, to some extent, collected “one operon at a time” where the attention of the research community is focused on one operon before moving to the next one. There is an approximately exponential decrease (see fit) in the reported number of operons as a function of the number of their regulatory interactions. To see if the binding site multiplicity profiles differ between global TFs and specific TFs we show in S2 Fig. the profiles for these two groups separately, but find no significant differences. It is perhaps surprising that even for such a well studied organism as *E. coli* more than half of the genes still lack any regulatory annotation. Among these unannotated genes we find important examples such as the genes responsible for mechanosensation *mscS, mscL, mscK, ynal, ybio* and *ybdG*. Preliminary results from our lab based on the method of Sort-seq [13] show that at least some of these genes might in fact be regulated. Other notable genes lacking regulatory annotation include: *lpp*, a lipoprotein believed to be one of the most abundant proteins in *E. coli* [33]; *rep*, a helicase required for genomic replication [34]; *kdpD* and *nhaB*, genes related to regulation of potassium [35] and sodium [36] levels in the cell. Nevertheless, it is still clear that many genes in *E. coli* do not strictly depend on TFs to be transcribed. This is in contrast with eukaryotic transcription where general TFs are necessary for the promoter recognition and transcription initiation process.

**Figure 4:**
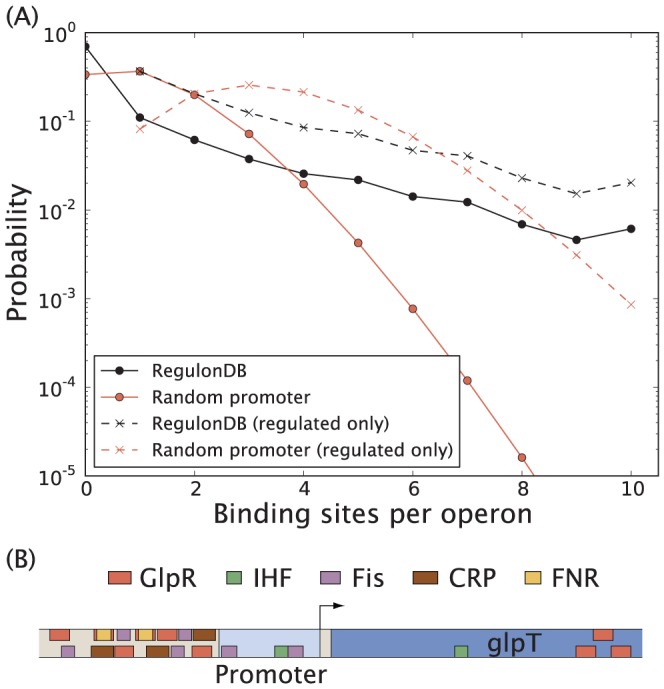
Number of TF binding sites per operon. (A) Distribution of number of TF binding sites per operon in RegulonDB 8.5 and the random promoter architecture model. Shown separately are distributions after excluding unregulated operons (“regulated only”). (B) The *glpTQ* operon is regulated by 21 binding sites for five different TFs.



We can compare the observed distribution of number of TF interactions per operon with the random promoter architecture model [see Models]. Looking at Fig. 4(A) we see some notable differences between the random promoter architecture model and the observed distribution. A larger number of operons are reported as unregulated in RegulonDB 8.5 than expected from the random promoter architecture model. Some TFs tend to bind to multiple sites per operon, which could result in a higher number of unregulated operons as compared to the random architecture model. We will address this in more detail below. Another explanation for the high number of unregulated operons could simply be that RegulonDB 8.5 is inherently biased and reports a higher fraction of unregulated operons than the actual value. The logic behind this hypothesis is that those operons for which there are known binding sites correspond in general to those that have been studied carefully, whereas many operons with no annotated binding sites simply have not been studied in detail. To consider the later possibility we modified the random promoter architecture model to exclude operons with no known regulatory interactions. In this case we update the prediction of the random promoter architecture model [Eq (2)] by first assigning one binding site to each of 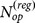 regulated operons. Then we randomly distribute the remaining 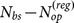 binding sites on the 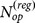 operons, as in Eq (2), leading to

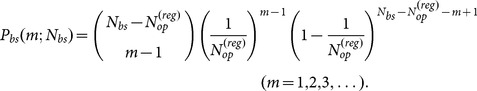
(10)


In Fig. 4(A) we now observe an overrepresentation of operons regulated from a single binding site, compared to the random promoter architecture model (compare black and blue dashed lines). This supports the idea that *E. coli* generally favors simple regulatory strategies when possible. In S3 Fig. we show that this conclusion holds separately for both global TF and specific TF binding sites. There is also a small group of highly regulated operons. For example *gadAXW*, coding for genes in the acid resistance system [37], is regulated by 35 TF binding sites. The operon *csgDEFG*, coding for genes that regulate the assembly and transport of extracellular amyloid fibres (known as Curli) [38], is regulated by 33 TF binding sites. Finally the operon *glpTQ*, shown schematically in Fig. 4(B), coding for genes responsible for the uptake and processing of glycerol-3-phosphate [39]–[41], is regulated by 21 binding sites for five different TFs. These promoter architectures could virtually never (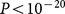, Eq. (2)) occur in the random promoter architecture model, and might as such be of interest for further study.

**Figure 5:**
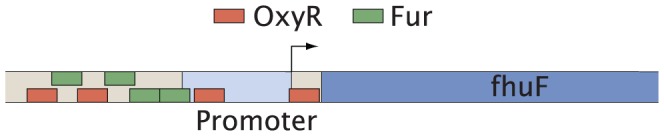
*fhuF* regulation. The TF OxyR is enriched for regulating genes at multiple binding sites as shown in Table 2. For example *fhuF* is regulated by four binding sites for OxyR.

We can also use the random promoter architecture model to study the number of TF interactions per operon for particular TFs. We expect this number to be higher than suggested by the random promoter architecture model since a TF can, for example, regulate an operon cooperatively from multiple sites. As an example the well-studied Lac repressor has three known binding sites in *E. coli* [2], all regulating the same operon (*lacZYA*). Had these three sites been randomly distributed over all operons, it would have been an unlikely outcome for them all to regulate the same operon. In Table 2 we show the number of operons regulated at multiple binding sites for a given TF, both in RegulonDB 8.5 and as predicted by the random promoter architecture model [Eq. (5)]. Many of these TFs differ very significantly from the random promoter architecture model, which could be indicative of multiple TF binding domains (e.g. OxyR [42], ArgR [43]), cooperative binding (e.g. TyrR [44]), TFs which repress operons by DNA looping (e.g. NagC [45]), or chromosomal restructuring through repeated TF binding (e.g. Fis [46]). In Fig. 5 we show the specific example of OxyR regulating the *fhuF* gene at four different binding sites. Interesting exceptions include Rob and MarA, which despite being common regulators do not bind to multiple binding sites at a single operon. Thus the random promoter architecture model allows us to identify TFs of special interest.

**Table 2:** TFs which are significantly enriched for multiple binding sites per operon, compared to the random promoter architecture model.

TF	Total number of binding sites	Operons regulated by multiple binding sites (RegulonDB)	Operons regulated by multiple binding sites (random promoter)	*p*-value
OxyR	44	19	0.69	1.9×10^−31^
ArgR	34	15	0.41	3.4×10^−27^
NarP	21	10	0.16	7.7×10^−22^
NarL	98	25	3.3	1.4×10^−19^
Fis	237	52	18	2.7×10^−17^
TyrR	19	8	0.13	2.0×10^−16^
FlhDC	30	10	0.32	1.0×10^−15^
IHF	114	25	4.5	1.5×10^−15^
CRP	320	67	31	3.5×10^−14^
CytR	23	8	0.19	4.8×10^−14^
NagC	23	8	0.19	4.8×10^−14^

The *p*-value for data in RegulonDB 8.5 is given by the probability of an equal or more extreme outcome in the random promoter architecture model. The particular example of OxyR regulating the *fhuF* at four binding sites is shown in Fig. 5. An extended version of Table 2, covering 115 TFs, is available in S2 Table.

**Figure 6:**
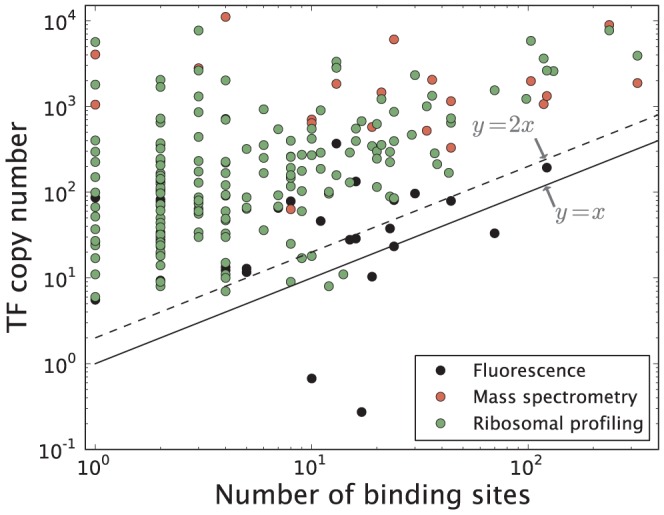
TF copy number plotted as a function of the total number of TF binding sites (RegulonDB 8.5) for that particular TF. The TF copy number is measured in [49]–[51]. The two lines mark the critical boundary where the number of binding sites is large enough to deplete TFs binding as monomers (solid) or dimers (dashed). Updated version of figure published in [47].

With a large number of targets we expect global TFs to be more abundantly expressed in the cell, to avoid running the risk of depleting the reservoir of TFs and hence the TF losing its ability to function effectively [47], [48]. In Fig. 6, we explore the relationship between TF copy number and corresponding number of binding sites, using three different genome-wide protein copy number censuses based on fluorescence measurements [49], mass spectrometry [50], and ribosomal profiling [51]. We find a statistically significant positive correlation in the data set based on ribosomal profiling (log-log slope = 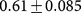), but not in the data sets based on fluorescence measurements (log-log slope = 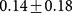) or mass spectrometry (log-log slope = 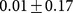). Here, we estimate the uncertainty in the linear fit parameter using the method of bootstrapping [52]. Large systematic deviations in the protein censuses [S1 Fig.] makes them difficult to use as means for model testing.

**Figure 7:**
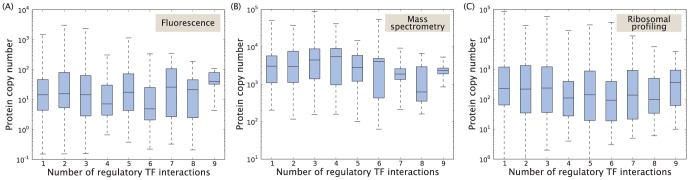
Measured protein copy number vs. number of TF binding sites regulating the transcription of the protein. The boxes show median, upper and lower quartiles, and the dashed lines show the range of the data. Protein data based on (A) fluorescence measurements [49], (B) mass spectrometry [50], and (C) ribosomal profiling [51].

Naively one could also imagine highly expressed genes to be subject to more regulation, because expressing too many of these would be energetically costly and expressing too few could have serious consequences to the fitness of the cell. By combining binding site multiplicities from RegulonDB 8.5 with the same protein copy number censuses [49]–[51] we can explore the possible relationship between these two quantities. In Fig. 7 we show the number of protein copies of a gene product as a function of the number of TF binding sites regulating the gene's expression (RegulonDB 8.5). The fluorescence based census shows a weak positive relation (log-log slope = 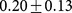) between these two magnitudes, the mass spectrometry census shows no significant relation (log-log slope = 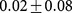), while the census based on ribosomal profiling presents a statistically significant negative relation (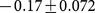). Again, the disagreement between the three protein censuses, shown in S1 Fig., makes it difficult to draw any definite conclusion regarding the relationship between gene regulation and protein expression and demonstrates a need for more rigor in the quantitative analysis of these problems.

### How are activator and repressor binding sites configured?

Many genes need to be expressed only under conditions satisfying some “combinatorial rule”. For example the *β*-galactosidase enzyme LacZ in *E. coli*, which cleaves lactose, is only highly expressed if lactose is present and glucose, the more favored energy source, is not present [53]. A general combinatorial promoter is regulated by one or more activators and repressors. Such combinatorial control requires multiple TFs to either activate and repress a gene, and the configuration of the two types of interactions determines the regulatory response. In this section we will study promoter architectures in more detail and their influence on gene expression.

**Figure 8:**
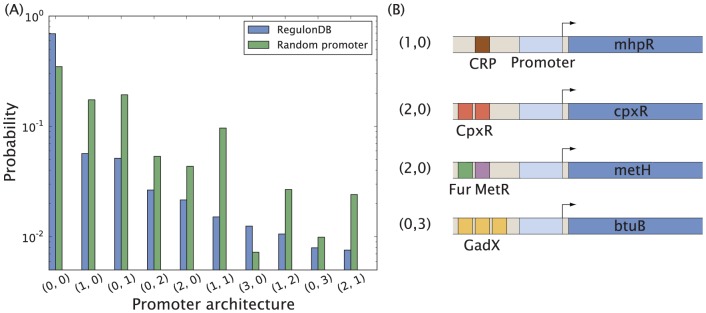
Frequency of promoter architectures. (A) Frequency of the most dominant promoter architectures listed in RegulonDB 8.5, and their corresponding frequency in the random promoter architecture model. Binding site configurations with *A* activator and *R* repressor binding sites are denoted by (*A,R*). (B) Examples of some of the architectures featured in (A).

To classify promoter architectures we adopt the notation 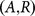 for a promoter regulated by 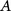 activator and 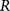 repressor binding sites. Using RegulonDB we can easily find the distribution 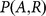 for 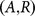 with respect to all known regulatory interactions in *E. coli*. We show the most dominant promoter architectures and some specific examples in Fig. 8, along with their expected frequency in the “two-TF” random promoter architecture model described in Models [Eq. (3)]. We see an almost equal use of repressors and activators, 53% vs. 47% interactions, and for each promoter architecture 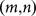 shown in Fig. 8(A) its symmetric counterpart 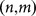 is almost equally present, both in absolute numbers and compared to the random promoter architecture model. Using the random promoter architecture model we can identify TF pairs which coregulate operons more frequently than one would expect by chance, a possible sign of TF-TF interactions or two TFs with otherwise related biological function [25]. In Table 3 we list the ten most such overrepresented TF pairs. The top pairs are all possible pairwise combinations of the MarA, SoxS and Rob TFs. These TFs are all paralogous proteins, having around 45% identical amino acid sequence at their N-terminals [54], responsible for regulating various stress responses. Note that some TFs might recognize similar or identical DNA sequences. In fact, this is the case of SoxS, Rob and MarA [55], and GalR and GalS [56]. FNR and ArcA are both global regulators responding to the availability of oxygen [57] in the cellular environment. NarL and NarP are homologous proteins responding to availability of nitrate and nitrite [58], and have been shown to act (anti)cooperatively with FNR [59], [60]. Fur and IHF are also global regulators, whose interplay with FNR has been investigated in [61]–[63]. GalR-GalS are homologous proteins responding to galactose [64], and GadX-GadW are homologous proteins responding to variations in pH level [37]. Even though TF pairs like Fis-CRP are more frequent coregulators (at 38 operons) in absolute numbers than any of the TF pairs listed in Table 3, this pair is not particularly overrepresented when compared to the random promoter architecture model (*p*-value “only” 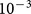), and their frequent coregulation can simply be attributed to the large number of CRP and Fis binding sites. Hence the random promoter architecture model allows us to find the most interesting TF pairs [Table 3].

**Table 3:** TF pairs which show significant enrichment for coregulation of operons, compared to the random promoter architecture model.

TF 1	TF 2	Total binding sites (TF 1)	Total binding sites (TF 2)	Coregulated operons (RegulonDB)	Coregulated operons (random promoter)	*p*-value
MarA	SoxS	24	29	18	0.26	5.5×10^−34^
MarA	Rob	24	17	14	0.15	1.2×10^−28^
SoxS	Rob	29	17	14	0.18	4.6×10^−27^
FNR	ArcA	131	118	30	5.3	6.4×10^−16^
FNR	NarL	131	98	27	4.5	3.0×10^−15^
NarP	NarL	21	98	11	0.75	1.7×10^−11^
FNR	Fur	131	122	24	5.5	2.8×10^−10^
FNR	IHF	131	114	23	5.2	4.3×10^−10^
GalR	GalS	12	12	5	0.054	5.5×10^−10^
GadX	GadW	37	20	6	0.27	1.5×10^−7^

The *p*-value for data in RegulonDB 8.5 is given by the probability of an equal or more extreme outcome in the random promoter architecture model. An extended version of Table 3, covering over 900 TF pairs, is available in S3 Table.

**Figure 9:**
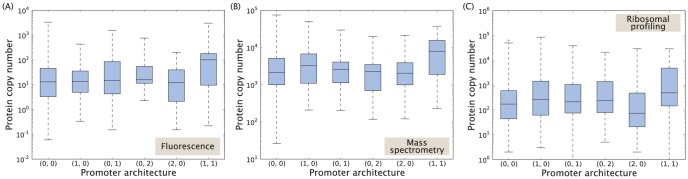
Protein copy number as a function of promoter architecture for the most common architectures. The notation (*A,R*) represents a promoter with *A* activator and *R* repressor binding sites. Protein data based on (A) fluorescence measurements [49], (B) mass spectrometry [50], and (C) ribosomal profiling [51].

Having identified the most common promoter architectures we are curious to find out how these relate to gene expression. Is there any relationship between promoter architecture and gene expression level for steady-state growth? To answer this question we identify all genes corresponding to a certain promoter architecture 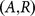 in RegulonDB and acquire the protein copy number distribution of these genes from the three *E. coli* protein censuses [49]–[51] [see Fig. 9]. Perhaps surprisingly, we find no systematic correlation between the number of activator and repressor binding sites, and gene expression in the three sets covering thousands of genes. The only exception is the promoter architecture with one activator and one repressor binding site each (1,1), whose median expression level is higher than the upper quartile of the other five studied promoter architectures, indicating that genes with this architecture might be more abundantly expressed. Still, the figure shows that even for a given promoter architecture there is a vast spread in protein copy number, spanning up to three orders of magnitude. It seems likely that all promoter architectures in Fig. 9 would be capable of producing proteins across the full range of biologically relevant concentrations. The main purpose of activators appears not to be increasing the maximum possible expression of a gene but rather, together with repressors, modulating it around a certain mean level. This mean level can on the other hand be achieved through other mechanisms, such as the ribosomal binding sequence (RBS) or promoter strength, which we will discuss in a later section.

### Where are TF binding sites located?

There are many different ways in which TFs can regulate the transcription rate of a gene. Perhaps most intuitively TFs can facilitate or block RNAP from interacting with a promoter of interest, to either activate or repress transcription of a gene [14]. However, TFs can modulate basically any step in the chain of events preceding promoter escape [65], or modify the DNA methylation or compactification states [66]. In eukaryotes, where the latter regulatory strategies are common, TF binding sites can be located hundreds of thousands of base pairs away from the transcription start site, which means that DNA needs to “loop” to establish a contact between TF and RNAP (if necessary for regulation) [66]. Hence each class of transcriptional regulation will have its own TF binding profile, and in this section we will investigate these profiles in more detail for *E. coli*.

**Figure 10:**
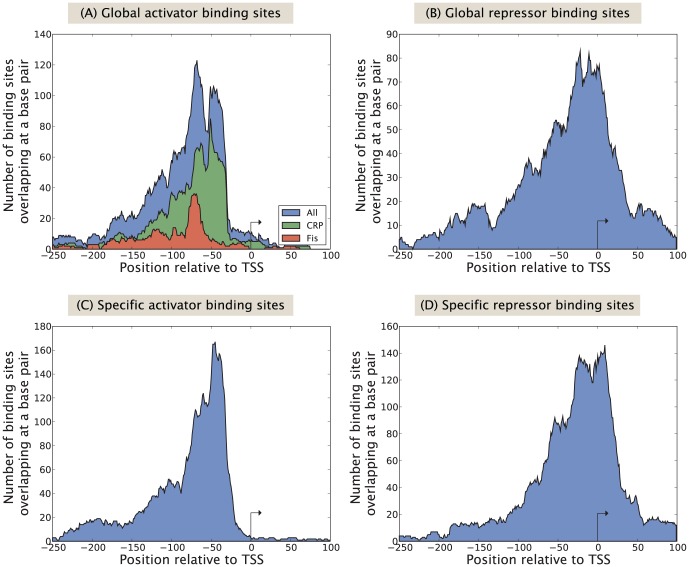
Distribution of activating and repressing binding sites bound by global TFs and specific TFs, respectively. The y-axis shows number of binding sites overlapping each nucleotide position, after aligning all promoters with respect to their transcription start site (TSS) for the different kinds of TFs. Similar figures were reported in [20] and [89] using earlier versions of RegulonDB.

After aligning all known promoters with respect to their transcription start site we can make a histogram [see Fig. 10] over the number of binding sites overlapping each nucleotide position. In eukaryotes, particularly in metazoans, DNA compaction through architectural complexes such as nucleosomes can bring TF binding sites in close physical proximity to promoters located millions of base pairs downstream [67]. As bacteria lack many of these architectural complexes, we hypothesize that binding sites in bacteria are constrained to be located closer to the promoter, leading to a narrower distribution of binding sites around the promoter as compared to eukaryotes. In fact 75% of all reported TF interactions in RegulonDB 8.5 take place within 100 bp of the transcription start site.

Activator and repressor binding sites have fundamentally different profiles; whereas repressors overlap the RNAP binding site for maximum repression, activators typically facilitate transcription initiation from upstream of the −35 region. TFs binding significantly upstream of −35 bp would, to a larger extent, need to loop DNA to interact directly with RNAP, or regulate expression of genes through other long range mechanisms. An interesting difference between specific activators [Fig. 10(C)] and global activators [Fig. 10(A)], is that the latter have two separate peaks, located at −70 bp and −45 bp respectively, rather than one. The TFs whose contribution dominates these two peaks, which should correspond to class I and class II activation [65], [68], are CRP and Fis (shown separately in Fig. 10(A)). Class I activators interact with the *α*-CTD domain, whereas class II activators interact directly with the sigma factor.

**Figure 11:**
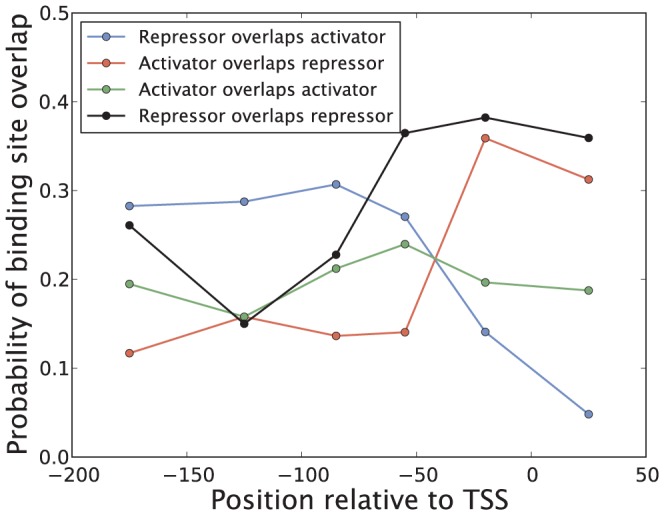
Probability of TF binding site overlap. Binding sites are defined as an interval of nucleotides from the 5×10^6^ bp *E. coli* genome covered by a TF upon binding. Two binding sites sharing one or more nucleotides are considered to be overlapping, independently of which strand the TFs bind. Binding sites overlapping more than one binding site are classified according to the site with the most overlap. Notice that the probabilities of “Repressor overlaps activators” and “Activator overlaps repressor” are not identical despite the number of overlapping activator and repressor binding sites in a region being fixed. For example, there are many more activators than repressors binding upstream of −100 bp, which results in a higher probability for a repressor to overlap with an activator in this region than vice versa.

Although most repressors function by blocking RNAP from binding the promoter, still roughly 25% of the repressors bind upstream of −70 bp, i.e. without the possibility of blocking RNAP [69]–[72]. Additional mechanisms through which an upstream repressor could block transcription is by forming DNA loops to contact the transcriptional machinery as well as downstream operators [9], [73]. Another possible way these upstream repressors could function is by preventing activators from binding the promoter, or inhibiting an activator from accessing its binding site without overlapping it via DNA allostery [10] or DNA bending [74], [75]. To test this hypothesis we show in Fig. 11 the probability of a repressor binding site overlapping an activator binding site as a function of position, using the probability for two activators to overlap as comparison. The results show that around 30% of the repressors binding upstream of −70 bp overlap with an activator, compared to 15–20% for two different activators. This suggests that blocking of activators is an important regulatory strategy for upstream repressors but not the only one, as a large fraction of upstream repressors inhibit transcription through other means.

In total, almost half of all binding sites reported in RegulonDB 8.5 overlap with other binding sites, which leads us to believe that this constitutes an important regulatory strategy. As more binding sites are discovered, the number of overlapping binding sites will likely increase, just as the probability of two students in a class having birthday on the same day goes up rapidly with the number of students. Interestingly, TFs often (37% of the reported overlapping interactions) overlap with themselves. For example, out of the 88 known Fur binding sites, 75% of them are reported to overlap with other Fur binding sites [76].

**Figure 12:**
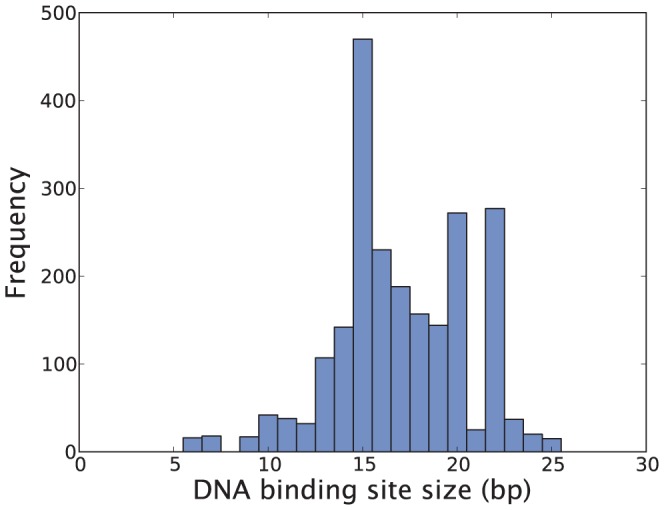
DNA binding site size (in base pairs) for all TF-DNA interactions (RegulonDB 8.5). Mean DNA binding site size size: 17.3 bp. Also see figure published in [77].

**Figure 13:**
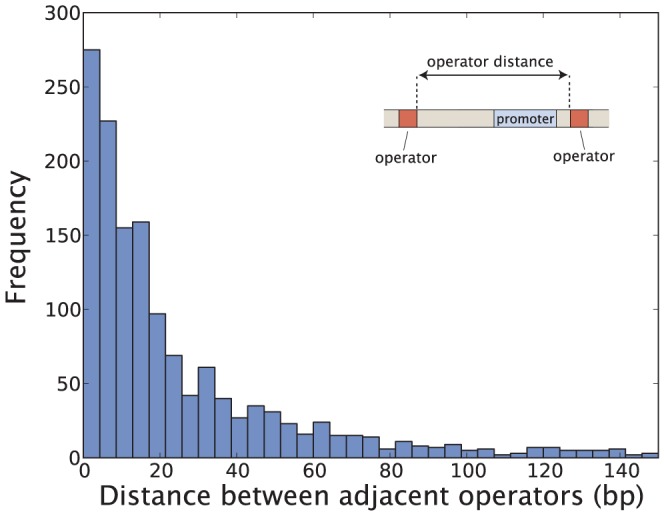
Edge to edge distance between adjacent binding sites (RegulonDB 8.5). Figure does not include binding sites separated by more than 150 bp, which would likely correspond to regulation of different operons.

Since the regulatory region of a gene is of limited size, TFs need to compete for space at promoters with other binding sites, in particular TFs which interact directly with RNAP. To study this “real estate” problem we first collect the DNA binding site size of all TF-DNA interaction sites reported in RegulonDB 8.5 [see Fig. 12]. A similar figure is reported in [77] using an earlier version of Regulon DB. Some of the notable peaks in Fig. 12 correspond mainly to binding of global TFs: Fis (15 bp), ArcA (15 bp) and CRP (22 bp). Most bacterial TFs interact with DNA along a contiguous region of around 15 bp (although outliers exist) which means that one could theoretically fit three nonoverlapping binding sites within 50 bp. Since the majority of operons reportedly have fewer than this number of binding sites [see Fig. 3], the size of the regulatory region does not in general seem to be a major constraining factor. However, for promoters with a larger number of binding sites, of which we saw some examples in Fig. 3, TFs would either need to bend DNA to access RNAP, or overlap with other TFs. To further study the real estate of the promoter we look at the separation between binding sites [see Fig. 13], which shows the the edge-to-edge distance for nonoverlapping adjacent binding sites. The majority of binding sites in this set are separated by less than 15 bp from their neighbors. Hence for an operon with three binding sites the regulatory region would be expected to take up around 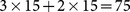 bp, around the same as observed in Fig. 10.

### How does promoter architecture relate to promoter strength?

Many prokaryotic genes do not rely on TFs for regulation, and will be constitutively expressed independently of the cellular environment. The production of these genes will, at our current best understanding, only be affected by the global availability of RNAP, sigma factors, ribosomes and the interaction strengths with these different complexes [78]. For proteins which are in constant demand, constitutive expression provides a simple and efficient choice of promoter architecture. Despite its simplicity, constitutive expression allows for an impressive dynamic range in protein production [26], as is also suggested by Fig. 9. This demonstrates the power of the basal production machinery, whose transcriptional component we will study further in this section. In particular we will be interested in the relationship between basal promoter strength and regulation by TFs.

**Figure 14:**
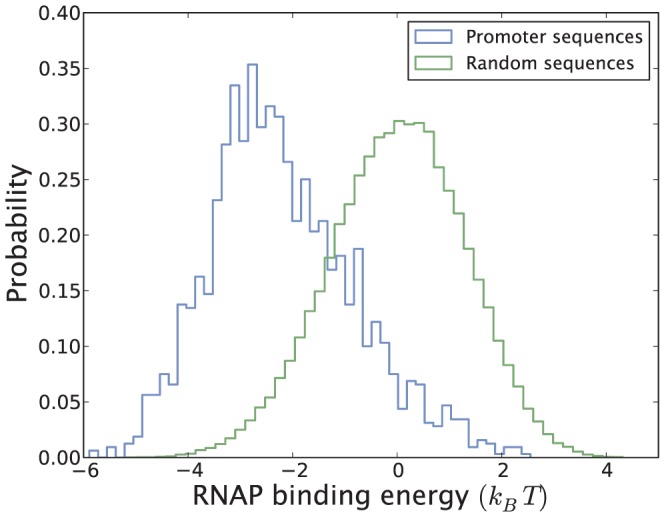
Predicted RNAP binding energy[26] for promoters in RegulonDB 8.5 and DNA sequences randomly chosen from the *E. coli* genome. The spacer region is allowed to range from 16–18 bp, and the −10 box is allowed to deviate by one base pair upstream or downstream from its consensus position. The RNAP binding energy is taken as the minimum binding energy of these 3×3 = 9 possible binding configurations.

**Figure 15:**
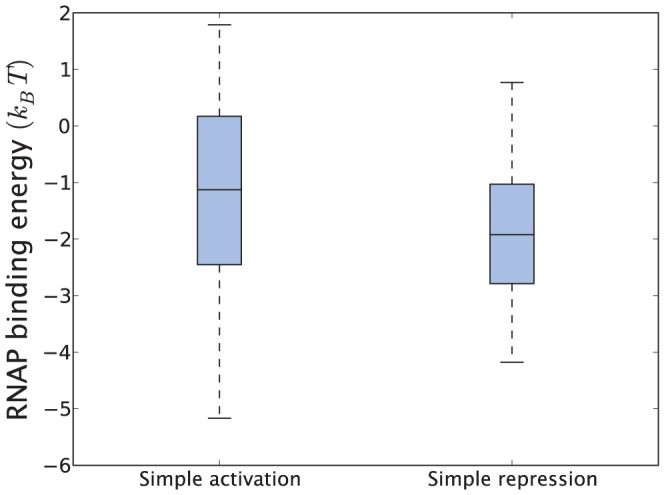
Predicted RNAP binding energy to promoters in the simple activation (1,0) and simple repression architecture (0,1). These calculations represent the basal state in which no TFs are present. Operons whose transcription is initiated from multiple promoters are excluded.

In *E. coli* the transcription rate of a gene can vary by up to three orders of magnitude due to differences in the promoter strength alone [26], not taking TFs into account. To illustrate this point we use the linear RNAP-DNA interaction model introduced in Models to predict the binding energy to all known 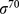 promoters along with the corresponding distribution for nonspecific binding [see Fig. 14]. As expected we get two separate distributions, where RNAP binds on average 2.4 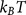 more strongly to known promoters than sequences chosen randomly from the *E. coli* genome. The predicted RNAP binding energy distribution spans roughly 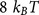 from the strongest to the weakest promoter, corresponding to a predicted 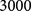-fold difference in RNAP binding affinity. This difference is similar to that found between the most abundantly expressed TFs (e.g. CRP) and scarcely expressed TFs (e.g. LacI) [79], [80] in *E. coli*, suggesting that promoter strength alone might be a powerful enough tool to set the mean level of gene expression to most biologically relevant values. Analysis of promoter sequences has revealed that functional transcriptional start sites are surrounded by noninitiating pseudopromoters [86]. A discomforting observation from Fig. 14, however, is that 200,000 sites or so in the 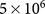 bp *E. coli* background interact more strongly with RNAP than the typical promoter. This raises several important questions [81]–[83]: Is the linear energy model missing key information, or can all the predicted promoters in principle produce transcripts? Do weak promoters need to be activated by TFs to function? Although trying to solve these important questions falls outside the scope of the current paper, we note that the paradox might originate from the fact that the promoter sequence encodes detailed information about both RNAP binding affinity, open complex formation rate and promoter escape rate [84], [85] in a way that likely cannot be captured in a simple linear model. Powerful new methods such as RNA-seq [87] could provide further insight into which of the 200,000 predicted promoters are actually transcriptionally active. In Fig. 9 we learned that the number of activator or repressor binding sites did not seem to have any systematic effect on the average gene expression in three sets covering thousands of genes. Since activators, by definition, increase the expression of a gene and repressors reduce it, the only possible explanation for this observation (if true) is that repressed genes have a higher basal level of expression. This could, for example, be the result if repressed genes have a higher affinity (*promoter strength*) for RNAP to their promoters. Since stronger promoters recruit RNAP more easily they would hence become transcriptionally more active. To investigate the relationship between promoter strength and promoter architecture we show in Fig. 15 the RNAP binding energy distribution for genes which according to RegulonDB 8.5 are regulated from a single activator or repressor binding site. For these promoters our data suggest, though not conclusively, that RNAP binds more strongly to the promoters of repressed genes, 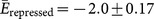 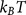, than promoters of activated genes, 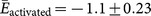 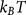. The reported uncertainty in the means are estimated using the method of bootstrapping [52]. Our results suggest that repressed genes have a higher basal rate of transcription, providing a possible explanation as to why we do not see a significant difference in gene expression as compared to activated genes. Conversely, weak promoters are more likely to be activated by TFs, suggesting that these promoters might not work effectively without TF activation.

## Discussion

After more than half a century of intense study *E. coli* remains one of the most important model organisms in biology. In order to make the vast pool of knowledge obtained from these studies publicly available and directly accessible, ambitious initiatives such as RegulonDB have curated thousands of references to collect information relating to TF binding site locations, organization of transcriptional units, and more. Although this annotation process is far from complete, as more than half of the *E. coli* genes still lack any known regulation, we now have a better opportunity than ever to study regulatory interactions in detail.

In this study we have analyzed TF-DNA interactions reported in RegulonDB 8.5. We find distinct differences in binding site location trends depending on TF type: activator or repressor, global or specific TFs. To study promoter architectures in greater depth we created a random promoter architecture model. This random model makes it possible to generate “null hypotheses” for promoter architectures which can then be compared to real regulatory architectures from RegulonDB data. Our findings can be summarized as follows:

1. We find that most promoters in *E. coli* are less heavily regulated than expected from the random promoter architecture model. The majority of operons in RegulonDB 8.5 have fewer than three known associated TF binding sites, and most specific TFs regulate fewer than three operons, suggesting that many *E. coli* genes are expressed with little “oversight” from TFs. Some interesting exceptions include operons such as gadAXW (acid resistance), csgDEFG (Curli amyloid fibers), and glpTQ (glycerol-3-phosphate uptake) which are controlled by up to 30 binding sites.
2. The random promoter architecture model allows us to identify, with well defined statistical significance, pairs of TFs which frequently coregulate operons, e.g. due to cooperative interactions or recognition of similar consensus sequences. Examples include the stress regulators MarA/Rob/SoxS, and the oxygen responding TFs FNR/ArcA. The random model further allows us to recognize TFs such as OxyR and Fis, which frequently bind to multiple binding sites per operon, e.g. due to cooperative binding, DNA looping, or through multiple binding domains. Our method of comparing promoter architectures to a null hypothesis provides a new approach for detecting coregulation and allows us to formulate experimentally testable hypotheses using only a list of known TF binding sites regulating each operon.
3. We find no systematic correlation between the number of activating or repressing TF interactions and the mean expression of a gene, as measured by three different genome-wide protein censuses covering thousands of genes. A position-weight-matrix model used to estimate the binding affinity of RNAP to promoters of activated and repressed genes, suggested that this lack of correlation might in part be due to differences in basal transcription rates of promoters. In this scenario, promoters that are being repressed have a higher basal level than promoters that are being activated.

One of the grand challenges of physical biology is to be able to construct predictive maps between promoter nucleotide sequence and gene expression. Increasingly accurate promoter architecture data, found e.g. using powerful techniques like ChIP-Seq, allow predictive maps to be both tested and refined. A difficulty with mapping promoter architecture and gene expression, apart from lacking complete knowledge of the regulatory network, is a substantial disagreement on protein concentrations as measured using different experimental methods and under different experimental conditions. The protein copy numbers measured using mass spectrometry [50] are for example on average at least one order of magnitude higher than for the same proteins measured with fluorescence based techniques [49], though these kinds of effects can be due to different growth conditions for the cells [78], [88]. As TF copy number plays a central role in regulatory function, we believe resolving these discrepancies will be a necessary step for a deeper understanding of several important aspects of gene regulation. To become quantitatively predictive, gene regulatory maps must come to relate gene expression data with precise promoter architecture data such as binding site locations and binding energies. These will allow for an accurate *in silico* description of global promoter activity, and provide quantitative predictions for genome-scale experiments.

## Supporting Information

S1 Fig **Comparison of different** ***E. coli*** **protein censuses.** Measured protein copy number using mass spectrometry [50], fluorescence [49], and ribosomal profiling [51]. Note how all measurements show a systematic deviation with respect to each other. This deviation can be up to two orders of magnitude, corresponding to comparing mass spectrometry and fluorescence.
(EPS)

S2 Fig **Number of binding sites and TF types regulating each operon (RegulonDB 8.5) shown separately for global TF binding sites (black) and specific TF binding sites (**red**)**.
(EPS)

S3 Fig **Number of TF binding sites per operon.** Distribution of number of TF binding sites per operon in RegulonDB 8.5 for (A) Global TF binding site and (B) Specific TF binding sites. Shown separately are distributions after excluding unregulated operons (“regulated only”).
(EPS)

S1 Table **All TFs and their associated number of binding sites, the number of operons regulated, and the total number of genes (coding sequences) regulated by each TF (RegulonDB 8.5).**
(CSV)

S2 Table **TFs which are significantly enriched for multiple binding sites per operon, compared to the random promoter architecture model.**
(CSV)

S3 Table **TF pairs which show significant enrichment for coregulation of operons, compared to the random promoter architecture model.**
(CSV)
