# Correction: The Influence of Promoter Architectures and Regulatory Motifs on Gene Expression in *Escherichia coli*

**DOI:** 10.1371/journal.pone.0121935

**Published:** 2015-03-06

**Authors:** The PLOS ONE Staff

There is an error in the legend for Fig. 2, “Number of operons, genes and binding sites regulated per TF (RegulonDB 8.5.).” Please see the completed, corrected Fig. 2 here.

**Fig 2:**
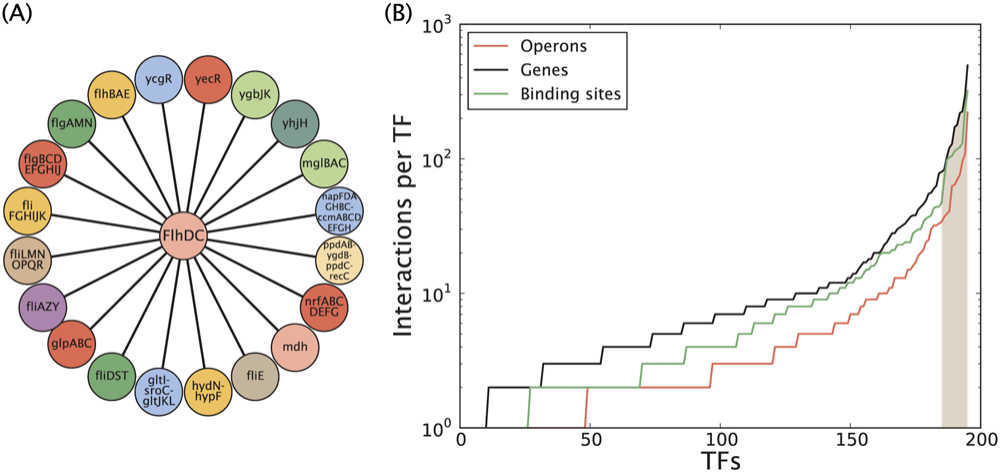
Number of operons, genes and binding sites regulated per TF (RegulonDB 8.5.). (A) Schematic of operons regulated by the FlhCD TFs according to RegulonDB 8.5. (B) The TFs have been sorted by increasing number of interactions, and the dark shaded area highlights the TFs responsible for 50% of all regulatory interactions in *E*. *coli*, which we denote as global TFs. The median number of operons, genes (coding sequences) and binding sites regulated per TF is 3, 6.5 and 4, respectively. The number of regulated genes is calculated by taking into account how many coding sequences are contained within each operon.
